# Epidemiology of Recurrent Hand, Foot and Mouth Disease, China, 2008–2015

**DOI:** 10.3201/eid2403.171303

**Published:** 2018-03

**Authors:** Jiao Huang, Qiaohong Liao, Mong How Ooi, Benjamin J. Cowling, Zhaorui Chang, Peng Wu, Fengfeng Liu, Yu Li, Li Luo, Shuanbao Yu, Hongjie Yu, Sheng Wei

**Affiliations:** Huazhong University of Science and Technology, Wuhan, China (J. Huang, S. Wei);; Chinese Center for Disease Control and Prevention, Beijing, China (J. Huang, Q. Liao, Z. Chang, F. Liu, Y. Li, L. Luo, S. Yu, H. Yu);; Sarawak General Hospital, Kuching, Malaysia (M.H. Ooi); Universiti Malaysia Sarawak, Kota Samarahan, Malaysia (M.H. Ooi);; The University of Hong Kong, Hong Kong, China (B.J. Cowling, P. Wu, Y. Li);; Fudan University, Shanghai, China (H. Yu)

**Keywords:** hand foot and mouth disease, recurrence, reinfection, enteroviruses, China, enterovirus A71, coxsackievirus A16, EV-A71, CV-A16, epidemiology, viruses

## Abstract

Using China’s national surveillance data on hand, foot and mouth disease (HFMD) for 2008–2015, we described the epidemiologic and virologic features of recurrent HFMD. A total of 398,010 patients had HFMD recurrence; 1,767 patients had 1,814 cases of recurrent laboratory-confirmed HFMD: 99 reinfections of enterovirus A71 (EV-A71) with EV-A71, 45 of coxsackievirus A16 (CV-A16) with CV-A16, 364 of other enteroviruses with other enteroviruses, 383 of EV-A71 with CV-A16 and CV-A16 with EV-A71, and 923 of EV-A71 or CV-A16 with other enteroviruses and other enteroviruses with EV-A71 or CV-A16. The probability of HFMD recurrence was 1.9% at 12 months, 3.3% at 24 months, 3.9% at 36 months, and 4.0% at 38.8 months after the primary episode. HFMD severity was not associated with recurrent episodes or time interval between episodes. Elucidation of the mechanism underlying HFMD recurrence with the same enterovirus serotype and confirmation that HFMD recurrence is not associated with disease severity is needed.

## Abstract 2

Children who have received the enterovirus A71 vaccine are still at risk for disease with infections of enteroviruses of other serotypes.

Hand, foot and mouth disease (HFMD) is a common childhood infectious disease that is mainly caused by enterovirus A71 (EV-A71), coxsackievirus A16 (CV-A16), and CV-A6 (*1*). Most HFMD patients exhibit a benign, self-limiting illness characterized by skin eruptions on the hands, feet, or buttocks and ulcers or blisters in the mouth with or without fever (*2*). However, some patients develop neurologic or cardiopulmonary complications or die (*3*,*4*). In the past 2 decades, outbreaks of HFMD have been documented in countries of the Western Pacific, including Malaysia, Japan, Singapore, Vietnam, and Cambodia (*5*–*9*). In China, HFMD has been prevalent since 2007. During 2008–2015, ≈13 million HFMD cases were reported, including 123,261 severe cases and 3,322 deaths in 31 provinces of mainland China.

Three inactivated monovalent EV-A71 vaccines have been licensed in China. Phase 3 clinical trials proved these vaccines had high efficacy (90.0%–97.4%) against EV-A71–associated HFMD (*10*,*11*) but did not confer cross-protection for HFMD caused by non–EV-A71 enteroviruses (*11*). A natural infection with EV-A71 also confers no or only short-term (<2 months duration) cross-protection against CV-A16–associated illness (*12*,*13*). Because of this limited cross-protection from infections of different enterovirus serotypes, multiple HFMD episodes can occur in a single person. Although observational studies indicate that the antibody response induced by the EV-A71 vaccine could last >2 years, reinfection with an enterovirus of the same serotype is still possible because the immunity induced by a natural enterovirus infection might not be lifelong (*14*). We accessed the national surveillance data for HFMD diagnosed during 2008–2015 in China, in an attempt to describe the epidemiologic features of patients with recurrent HFMD and examine the relationship between disease severity and HFMD recurrence.

## Materials and Methods

### Data Sources

As described previously (*1*), HFMD cases were reported voluntarily to the Chinese Center for Disease Control and Prevention (China CDC) during January 1, 2008–May 1, 2008, and starting May 2, 2008, cases were mandatorily reported online to China CDC within 24 hours after diagnosis. We collected information on basic demographics (name, sex, national identification number, date of birth, home address, name of either of the patient’s parents, contact telephone number); case classification (probable or confirmed); disease severity (severe or mild); date of illness onset, diagnosis, and death (if applicable); and enterovirus serotype (for confirmed cases). For virologic surveillance, clinical specimens were collected from a subsample of cases from each province and tested by PCR with primers and probes for panenterovirus, EV-A71, and CV-A16. We assumed that the enterovirus identified in HFMD patient samples was the causative enterovirus of the HFMD episode.

We included the HFMD surveillance data of 29 provinces of mainland China collected during January 1, 2008–December 31, 2015. We excluded data from Hunan and Hubei Provinces from this study because (since 2010 for Hunan Province and 2012 for Hubei Province) most of the hospitals in these provinces reported EV-A71 infection on the basis of EV-A71 IgM antibody detection assays, which are unreliable tests (*15*–*17*).

### Case Definitions

We defined a probable HFMD patient as a patient who had rashes on the hands, feet, mouth, or buttocks and ulcers or vesicles in the mouth with or without fever. We defined a laboratory-confirmed patient as a probable patient with laboratory evidence of infection with EV-A71, CV-A16, or other enteroviruses. The diagnostic tests used for enterovirus detection were reverse transcription PCR and real-time reverse transcription PCR. Patients were classified as having severe HFMD if they had any complications (i.e., aseptic meningitis, brainstem encephalitis, encephalitis, encephalomyelitis, acute flaccid paralysis or autonomic nervous system dysregulation, pulmonary edema, pulmonary hemorrhage, or cardiorespiratory failure). Otherwise, patients were classified as having mild HFMD.

**Figure 1:**
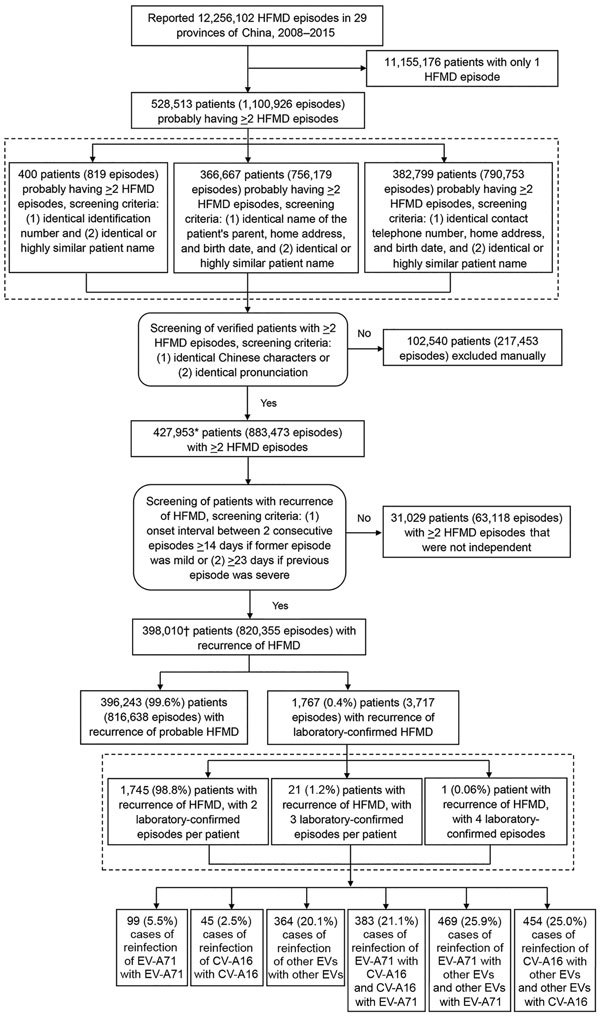
Flowchart showing screening for and analysis of patients with recurrent HFMD from the national HFMD surveillance database, 29 provinces of China, 2008–2015. Percentages do not equal 100% because of rounding. *The number of patients (427,953) with >2 HFMD episodes is higher than expected (528,513 – 102,540 = 425,973) because of improved patient matching. In some situations, the number of patients with >2 episodes did not change; for example, a patient initially identified with 3 episodes might have been determined to have only 2 episodes, with the third episode being attributed to a different patient. In other situations, the number of patients with >2 episodes decreased; for example, a patient initially identified as having 3 episodes might have been determined to be 3 different patients with 3 different episodes. Therefore, the reduced number of patients (528,513 – 427,953 = 100,560) with >2 HFMD episodes is smaller than the number of patients (102,540) excluded manually. †The number of patients (398,010) with recurrence of HFMD is higher than expected (427,953 – 31,029 = 396,924) because some patients needed to be excluded and included. In some situations, patients were completely included or excluded from the recurrent HFMD patient population sample; for example, all 3 episodes of a patient could have been determined to not be independent from each other. In other situations, patients were included and excluded from the recurrent HFMD patient population sample; for example, a patient with 3 episodes might have had 2 episodes that were not independent from each other. In these cases, the patient had 2 episodes included and 1 episode excluded; therefore, the number of included patients plus excluded patients (398,010 + 31,029 = 429,039) exceeded the starting population number (427,953). CV-A16, coxsackievirus A16; EV-A71, enterovirus A71; HFMD, hand, foot and mouth disease; other EVs, other non–EV-A71 and non–CV-A16 enteroviruses.

We identified patients with >2 episodes of HFMD reported in the national HFMD surveillance system by matching records using any of the following 3 screening criteria: 1) having identical identification number and identical or highly similar patient name; 2) having identical patient’s parent name, home address, and birth date and identical or highly similar patient name; and 3) having identical contact telephone number, home address, and birth date and identical or highly similar patient name (Technical Appendix). We considered patients to have recurrent HFMD if they experienced >2 independent episodes of HFMD. We defined independent episodes as consecutive episodes separated by an interval of >14 days if the previous episode was mild and >23 days if the previous episode was severe. We had estimated the time intervals defining 2 independent episodes by adding the longest duration of HFMD reported (7 days for mild illness and 16 days for severe illness) (*18*–*21*) plus the longest incubation period reported (7 days) (*4*,*22*–*26*). We classified patients with >2 independent episodes of laboratory-confirmed HFMD as having recurrent laboratory-confirmed HFMD. Otherwise, we classified patients as having recurrent probable HFMD. When counting the number of cases of reinfection with EV-A71, CV-A16, or other enteroviruses (Figure 1; Table 1), we considered any 2 laboratory-confirmed episodes as 1 case of reinfection; therefore, we classified patients with 3 laboratory-confirmed HFMD episodes as having 3 cases of reinfection (i.e., we grouped episodes 1 and 2, 1 and 3, and 2 and 3 together) and 4 laboratory-confirmed HFMD episodes as having 6 cases of reinfection (i.e., we grouped episodes 1 and 2, 1 and 3, 1 and 4, 2 and 3, 2 and 4, and 3 and 4 together).

**Table 1:** Demographic characteristics of patients with recurrent probable and laboratory-confirmed HFMD in 29 provinces of China, 2008–2015*

Characteristic	Patients with recurrent probable HFMD, N = 396,243	Cases of recurrent laboratory-confirmed HFMD, N = 1,814†						Patients with recurrent laboratory-confirmed HFMD, N = 1,767
Reinfection after EV-A71 with EV-A71, n = 99	Reinfection after CV-A16 with CV-A16, n = 45	Reinfection after other EVs with other EVs, n = 364	Reinfection after EV-A71 with CV-A16 or after CV-A61 with EV-A71, n = 383	Reinfection after EV-A71 with other EVs or after other EVs with EV-A71, n = 469	Reinfection after CV-A16 with other EVs or after other EVs with CV-A16, n = 454
Age at first episode								
Age, mo, median (IQR)	20.8 (12.2–31.4)	24.2 (15.6–36.5)	27.1 (20.9–39.4)	18.8 (12.2–31.4)	26.3 (17.7–36.8)	22.6 (14.5–34.4)	22.8 (14.2–32.8)	22.6 (14.2–34.0)
Age group								
<6 mo	7,279 (2)	1 (1)	0	10 (3)	1 (0.3)	4 (1)	7 (2)	23 (1)
6–11 mo	80,982 (20)	10 (12)	7 (16)	77 (21)	39 (10)	76 (16)	72 (16)	283 (16)
12–23 mo	155,973 (39)	46 (46)	14 (31)	144 (40)	132 (35)	181 (39)	176 (38)	696 (39)
24–59 mo	145,289 (37)	39 (39)	22 (49)	129 (35)	203 (53)	204 (43)	192 (42)	738 (42)
5–9 y	6,526 (2)	2 (2)	2 (4)	4 (1)	8 (2)	4 (1)	7 (2)	26 (2)
10–14 y	158 (0.04)	1 (1)	0	0	0	0	0	1 (0.05)
>15 y	36 (0.01)	0	0	0	0	0	0	0
Age at second episode								
Age, mo, median (IQR)	36.4 (24.3–48.5)	40.0 (27.4–50.2)	40.7 (25.7–55.8)	34.7 (24.2–45.9)	42.3 (32.5–53.2)	37.1 (26.8–49.4)	36.8 (26.8–49.2)	36.5 (25.7–48.7)
Age group								
<6 mo	236 (0.06)	0	0	1 (0.3)	0	0	0	1 (0.05)
6–11 mo	14,239 (4)	2 (2)	0	12 (3)	3 (1)	14 (3)	13 (3)	47 (3)
12–23 mo	83,568 (21)	17 (17)	8 (18)	89 (25)	35 (9)	73 (16)	73 (16)	309 (17)
24–59 mo	257,729 (65)	69 (70)	29 (64)	232 (64)	298 (78)	335 (71)	315 (69)	1,234 (70)
5–9 y	39,786 (10)	10 (10)	7 (16)	29 (8)	47 (12)	45 (10)	52 (12)	170 (10)
10–14 y	640 (0.16)	1 (1)	1 (2)	1 (0.3)	0	2 (0.4)	1 (0.2)	6 (0.33)
>15 y	45 (0.01)	0	0	0	0	0	0	0
Male sex	259,028 (65)	74 (75)	31 (69)	247 (68)	270 (70)	326 (70)	291 (64)	1,208 (68)
Rural residence	186,700 (47)	49 (49)	19 (42)	115 (32)	187 (49)	190 (41)	167 (37)	716 (41)
Frequency of episodes								
2	373,745 (95)	91 (92)	41 (91)	303 (83)	356 (93)	404 (86)	400 (88)	1,595 (90)
3	21,023 (5)	7 (7)	4 (9)	54 (15)	22 (6)	59 (13)	49 (11)	161 (9)
>4‡	1,475 (0.4)	1 (1)	0	7 (2)	5 (1)	6 (1)	5 (1)	11 (1)
Death	20 (0.005)	0	0	0	0	0	0	0

*Data are no. (%) patients unless otherwise indicated. CV-A16, coxsackievirus A16; EV-A71, enterovirus A71; HFMD, hand, foot and mouth disease; IQR, interquartile range; other EVs, other non–EV-A71 and non–CV-A16 enteroviruses. †For patients with laboratory-confirmed recurrence of 3 or 4 HFMD episodes, any 2 laboratory-confirmed HFMD episodes were combined to form a case of recurrence of laboratory-confirmed HFMD. For example, patients with 3 episodes were categorized as having 3 reinfections, with the first and second, first and third, and second and third infections being grouped together. ‡For patients with recurrence of laboratory-confirmed HFMD, the highest number of episodes was 4. For patients with recurrence of probable HFMD, the highest number of episodes was 8.

### Data Analysis

We used medians and interquartile ranges (IQRs) to describe continuous variables and numbers and percentages to summarize categorical variables. We used logistic regression with the forward stepwise selection approach to explore the association between HFMD recurrence and severe disease. We denoted the results as odds ratios (ORs) with 95% CIs. All statistical tests were 2-sided, and we considered an α of 0.05 statistically significant.

We defined the probability of HFMD recurrence as the probability of occurrence of HFMD among children who previously had HFMD and estimated recurrence using survival analysis (the Kaplan-Meier method). To calculate the probability of HFMD recurrence, we took only the event of interest into account and censored other events at the end of observation. When estimating the probability of HFMD recurrence, we enrolled all patients with recurrent HFMD (whether probable or laboratory-confirmed) in the analysis. We censored patients with only 1 HFMD episode. When estimating the probability of reinfection after EV-A71 with EV-A71, we included only the case-patients with a primary episode of EV-A71 infection who were later reinfected with EV-A71. We censored case-patients who had just 1 infection with EV-A71 (i.e., case-patients who were infected with EV-A71 then infected with CV-A16 or other enteroviruses, case-patients who were infected with EV-A71 then had probable HFMD, and case-patients with a single episode of EV-A71 infection). We used similar analyses to estimate the probability of reinfection with the same serotype for other serotypes of enterovirus.

We also conducted a sensitivity analysis to account for the uncertainty of the intervals used to define 2 independent HFMD episodes; in this analysis, we used 16 days (for previous mild episodes) and 61 days (for previous severe episodes) as cutoff values, which were derived from another investigation conducted in China that also investigated HFMD recurrence (*27*). We conducted data cleaning and analyses using R Project version 3.2.5 (http://cran.r-project.org) and ArcGIS 10.2 (http://www.esri.com/arcgis/about-arcgis). This study was approved by the ethics review committees of China CDC (Beijing, China).

## Results

**Figure 2:**
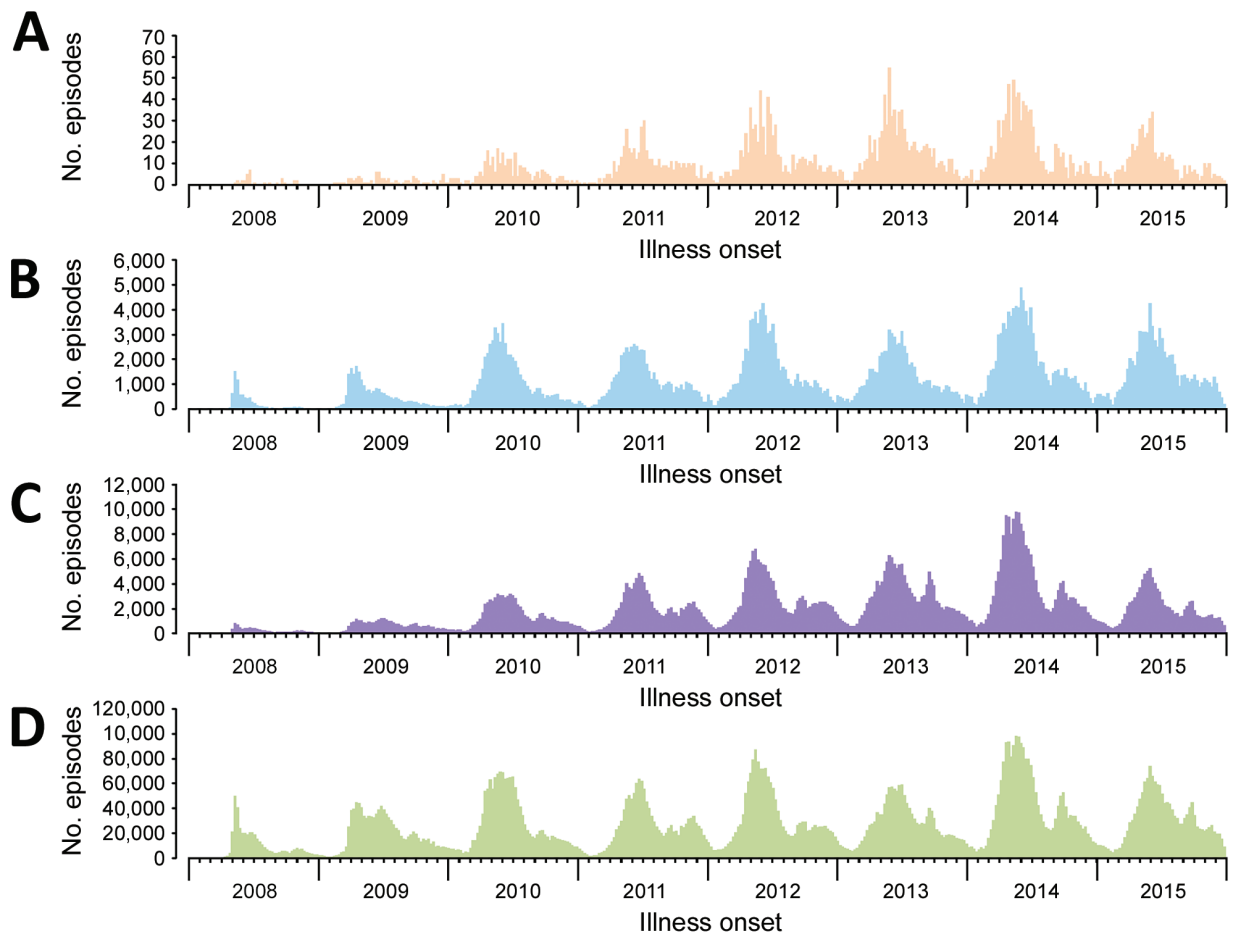
Hand, foot and mouth disease (HFMD) episodes in 29 provinces of China, 2008–2015. A) Patients with recurrent laboratory-confirmed HFMD. B) Patients with single episode of laboratory-confirmed HFMD. C) Patients with recurrent probable HFMD. D) Patients with single episode of probable HFMD.

During 2008–2015, a total of 12,256,102 HFMD episodes occurring in 29 provinces of China were reported to the China CDC surveillance system. When using >14-day and >23-day intervals for defining independent infections, 398,010 patients (having 820,355 [7%] episodes) were identified as having recurrent HFMD, of which 1,767 (0.4%) patients (having 3,717 episodes) had recurrent laboratory-confirmed HFMD (Figure 1). The number of patients with recurrent HFMD was similar when we repeated this analysis using the 16-day and 61-day cutoff values in the sensitivity analysis, indicating that our estimation of HFMD recurrence was robust (Technical Appendix Figure). Compared with patients with only 1 laboratory-confirmed HFMD episode, patients with recurrent laboratory-confirmed HFMD had a similar seasonal pattern, presenting semiannual peaks of activity with a major peak in the spring and early summer (April–June) followed by a smaller peak in autumn (September–October) (Figure 2, panels A, B). Similar seasonality was also observed for patients with a single episode of and recurrent probable HFMD (Figure 2, panels C, D).

We next focused on analyzing the 1,767 patients with recurrent laboratory-confirmed HFMD. During the study period, 90.3% (1,595) of these patients had 2 episodes and 9.7% (172) had >2 episodes: 161 (9.1%) patients had 3 episodes and 11 (0.6%) patients had 4 episodes. In total, 9% (154/1,767) of the patients with recurrent laboratory-confirmed HFMD still had >1 episode of probable HFMD, and 1,613 patients had only episodes of laboratory-confirmed HFMD. Of the 157 (8.9%) patients with >1 severe HFMD episode (183 total severe episodes), 26 patients (20 with 2 episodes, 3 with 3 episodes, and 3 with 4 episodes) experienced 2 severe HFMD episodes. A total of 1,814 cases of recurrence occurred among the 1,767 patients with recurrent HFMD. Only 144 (8%) of these 1,814 cases involved reinfection with an enterovirus of the same serotype: 99 (5.5%) with EV-A71 and 45 (2.5%) with CV-A16 (Figure 1). Most recurrent HFMD cases were caused by enteroviruses of different serotypes. Of the 1,767 patients, 5 (0.3%) were found to have an interval of <20 days between consecutive HFMD episodes: 2 patients who were reinfected with enteroviruses of different serotypes and 3 patients who were reinfected with enteroviruses of the same serotype.

### Demographic Characteristics

The median ages of patients with recurrent laboratory-confirmed HFMD were 22.6 (IQR 14.2–34.0) months for the first episode and 36.5 (IQR 25.7–48.7) months for the second episode. Younger children had more episodes of recurrent laboratory-confirmed HFMD (p<0.001) and recurrent probable HFMD (p = 0.001) (Technical Appendix Table 1). Few patients (1.5%) had their first episode of HFMD after 5 years of age. Approximately two thirds (68% or 1,208) of the patients affected were boys, and 41% were residents of rural areas. The demographic characteristics age, sex, and rural residence and the frequency of episodes were similar among patients with recurrent laboratory-confirmed HFMD, regardless of the enterovirus serotypes of reinfections (Table 1).

### Geographic Distribution

**Figure 3:**
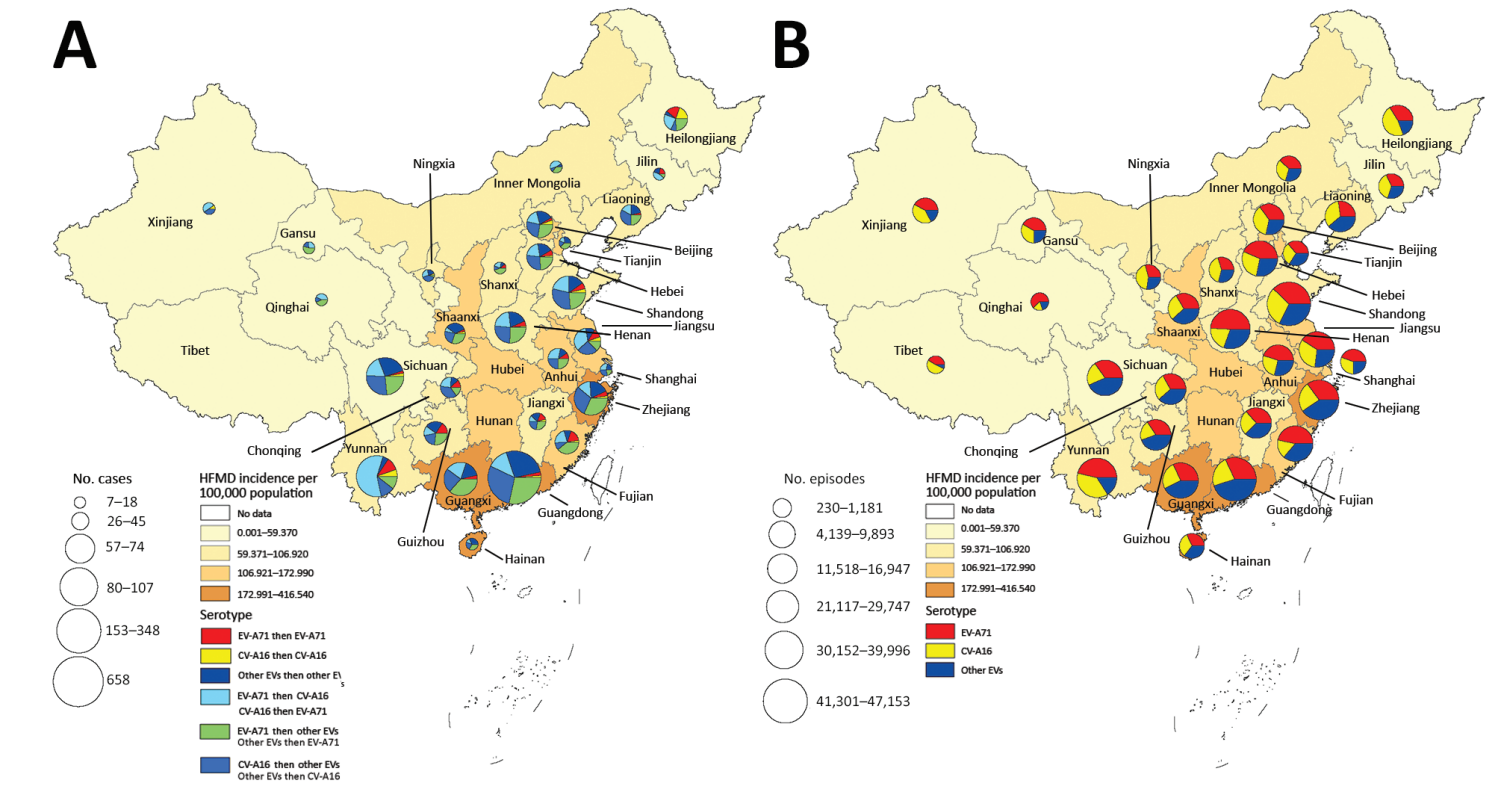
Geographic distribution of patients with recurrent HFMD (A) and episodes of enterovirus infection (B) in 29 provinces of China, 2008–2015. A) Pie charts correspond to the number of recurrent laboratory-confirmed HFMD cases. B) Pie charts correspond to the number of laboratory-confirmed HFMD episodes. CV-A16, coxsackievirus A16; EV-A71, enterovirus A71; HFMD, hand, foot and mouth disease; other EVs, non–EV-A71 and non–CV-A16 enteroviruses.

Patients with recurrent laboratory-confirmed HFMD were observed in all of the 29 provinces of China we examined except Tibet. The number of recurrent laboratory-confirmed HFMD cases varied substantially, ranging from 7 cases in Qinghai Province to 658 cases in Guangdong Province. Half of the cases with recurrent laboratory-confirmed HFMD were reported in 3 provinces: Guangdong (658 cases), Yunnan (153 cases), and Sichuan (107 cases) (Figure 3).

### Probability of HFMD Recurrence

**Figure 4:**
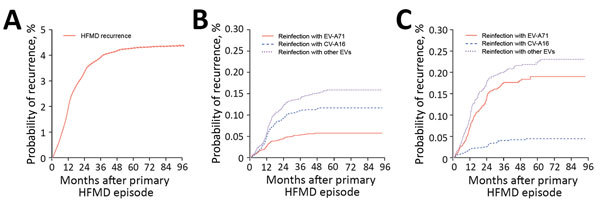
Kaplan-Meier analysis of survival from HFMD recurrence after primary HFMD diagnosis, 29 provinces of China, 2008–2015. A) Probability of HFMD recurrence among all patients who had probable and laboratory-confirmed HFMD. B) Probability of HFMD recurrence among case-patients whose primary episode was an infection with EV-A71. C) Probability of HFMD recurrence among case-patients whose primary episode was an infection with CV-A16. CV-A16, coxsackievirus A16; EV-A71, enterovirus A71; other EVs, non–EV-A71 and non–CV-A16 enteroviruses; HFMD, hand, foot and mouth disease.

Patients in this cohort were under observation for a median of 38.0 (range 0–97.4) months after their first HFMD diagnosis (Technical Appendix Table 2). The recurrent episode occurred 0.5–93.4 (median 11.7) months after the primary HFMD episode in patients with recurrence of probable HFMD and 0.5–62.1 (median 12.0) months in patients with recurrence of laboratory-confirmed HFMD. The probability of HFMD recurrence was 1.9% at 12 months, 3.3% at 24 months, and 3.9% at 36 months; however, recurrence remained at 4.0% at 38.8 months after the primary episode of HFMD (Figure 4, panel A). For patients with primary EV-A71 infections, the probability of reinfection with CV-A16 (0.11% [95% CI 0.10%–0.13%]) or other enteroviruses (0.14% [95% CI 0.13%–0.16%]) was higher than that of reinfection with EV-A71 (0.05% [95% CI 0.04%–0.07%]; p<0.001) (Figure 4, panel B). For patients with primary CV-A16 infections, the probability of reinfection with EV-A71 (0.18% [95% CI 0.15%–0.20%]) or other enteroviruses (0.21% [95% CI 0.18%–0.23%]) was higher than that of reinfection with CV-A16 (0.04% [95% CI 0.03%–0.05%]; p<0.001) (Figure 4, panel C). These findings suggest that risk for reinfection with different enterovirus serotypes might be higher than that for reinfection with identical enterovirus serotypes.

### Relationship between HFMD Recurrence and Severe Illness

Unsurprisingly, we found that illness severity was inversely associated with age and onset-to-diagnosis interval. EV-A71 infections (OR 7.2, 95% CI 4.0–13.0) and other enterovirus infections (OR 2.7, 95% CI 1.5–5.0) were more severe than CV-A16 infections. Patients who lived in urban areas also had increased risk for severe illness (OR 1.8, 95% CI 1.3–2.5). After adjusting for these risk factors, recurrent HFMD episodes were not found to be associated with illness severity, which means the second and third HFMD episodes did not appear to be more or less severe than the first episode. In addition, the interval between the 2 episodes was not related to disease severity of the latter episode (OR 0.97, 95% CI 0.95–1.01) (Table 2).

**Table 2:** Risk factors associated with severe illness in cases of recurrent laboratory-confirmed HFMD, China, 2008–2015*

Risk factor	Mild, N = 3,187, no. (%)	Severe, N = 172, no. (%)	Adjusted OR (95% CI)
Age at HFMD onset, mo			
>23	2,054 (96.9)	66 (3.1)	Reference
12–23	836 (91.5)	78 (8.5)	2.4 (1.7–3.6)
<12	297 (91.4)	28 (8.6)	2.6 (1.6–4.4)
Sex			
F	1,010 (95.5)	48 (4.5)	Reference
M	2,177 (94.6)	124 (5.4)	0.9 (0.6–1.2)
Enterovirus serotype			
CV-A16	857 (98.5)	13 (1.5)	Reference
Other EVs	1,452 (96.0)	61 (4.0)	2.7 (1.5–5.0)
EV-A71	878 (90.0)	98 (10.0)	7.2 (4.0–13.0)
Residence			
Rural	1,342 (92.8)	104 (7.2)	Reference
Urban	1,845 (96.4)	68 (3.6)	1.8 (1.3–2.5)
Order episode occurred			
First	1,523 (93.9)	99 (6.1)	Reference
Second or after	1,664 (95.8)	73 (4.2)	0.8 (0.5–1.2)
Onset-to-diagnosis interval			
<1 d	1,443 (96.3)	55 (3.7)	Reference
2–3 d	896 (94.0)	57 (6.0)	1.6 (1.1–2.4)
>4 d	848 (93.4)	60 (6.6)	1.8 (1.2–2.7)
Interval between successive episodes			0.97 (0.95–1.01)

*CV-A16, coxsackievirus A16; EV-A71, enterovirus A71; HFMD, hand, foot and mouth disease; OR, odds ratio; other EVs, other non–EV-A71 and non–CV-A16 enteroviruses.

## Discussion

During 2008–2015, we found that 398,010 HFMD patients with >2 episodes (a total of ≈820,000 episodes) were reported among children in China; 1,767 of these recurrences were laboratory-confirmed. The patients who were reinfected with different enterovirus serotypes had similar age, sex, residence, and frequency of episodes. Recurrence of HFMD mainly occurred 0–38.8 months after the primary episode, with a recurrence probability of 4% at 38.8 months. Reinfection with a different enterovirus serotype was more likely than reinfection with an identical enterovirus serotype. The severity of HFMD was not associated with recurrent infections or the time interval between HFMD episodes.

In a report on a phase 3 clinical trial, an EV-A71 neutralizing antibody titer of 1:16 was associated with protection against EV-A71–associated HFMD (*28*). In addition, EV-A71 was observed to confer cross-neutralization activity against major global EV-A71 genotypes (A, B1, B3–B5, C1–C5), although the degree of cross-neutralization varied (*29*–*31*). In an EV-A71 vaccine study, participants were observed for only 2 years, but results suggested that the vaccine can provide protection against EV-A71–associated HFMD for >2 years (*14*). Infection with enteroviruses seems to confer lifelong immunity to HFMD, given that adult cases are rare (*1*). The reasons underlying the cases of HFMD recurrence caused by reinfections with the same serotype, which have a 12-month median interval to reinfection, are not clear. Measles has been deemed to provide long-lasting protection against reinfection. However, measles reinfections have occurred in vaccinated and presumptively immune persons, either because of insufficient primary antigenic stimulation or inadequate duration of antibody response (*32*). Study results have suggested the involvement of multiple cellular deficiencies, including low memory B-cell count, reduced polyclonal naive and memory T-cell responses, and suboptimal antigen-presenting cell responses, in children with low vaccine responses (*33*,*34*). Agammaglobulinemia is another condition of immunodeficiency associated with recurrent infections (*35*). In patients with influenza, suboptimal immune responses after the primary infection led to the failure to generate a protective immune response that could have prevented reinfection (*36*). Children with immature immunity or immunodeficiency (*37*) probably are not able to induce sufficient immune responses (or might induce low-level serologic responses that wane rapidly) after infection with EV-A71 or CV-A16; thus, these children are likely susceptible to reinfection with an enterovirus of the same serotype as their primary episode. Another possibility (though less likely) is that high neutralizing antibody titers might not protect some persons from illnesses induced by enteroviruses. Further investigations are needed to provide a scientific explanation.

Even though the genotypes of EV-A71 and CV-A16 were not available in this study, previous studies have shown that the predominant EV-A71 and CV-A16 genotypes circulating in China have been consistent. Phylogenetic analysis of viral protein 1 gene sequences revealed that the EV-A71 genotype circulating in China since 2008 has been C4 (*38*–*42*); hence, the monovalent EV-A71 vaccines licensed in China were designed to target the C4 genotype. B1 has been reported to be the predominant genotype of CV-A16 circulating in China (*40*–*43*). Therefore, we reasonably conclude that in our cohort HFMD recurrences involving reinfections with enteroviruses of the same serotype were also highly likely reinfections with the same genotype.

Recurrent laboratory-confirmed HFMD was largely (at least 72% of cases) attributable to different serotypes of enterovirus; thus, undoubtedly hundreds of thousands of patients with HFMD recurrence with different serotypes should have occurred, given clinical samples were collected from only 4.2% of the patients with probable HFMD episodes for virologic diagnosis. Our results support the notion that limited or no cross-protection against different serotypes of enterovirus occurs after natural infection, which is consistent with observations from the EV-A71 vaccine study (*12*,*14*) and a modeling study of natural infections (*13*). Antibody-dependent enhancement, which has been commonly seen in dengue, was also observed in EV-A71 and CV-B3 infections in mouse models; the severity of the subsequent episode of infection was enhanced by a subneutralizing level of antibody after primary infection (*44*,*45*). However, we did not observe that HFMD recurrence or the interval between successive episodes had any effect on disease severity in humans. It seems that antibody-dependent enhancement does not happen in human infections with enterovirus, further implying that no or short-term cross-reactivity develops for different serotypes of enterovirus.

Three monovalent EV-A71 vaccines are administered in China (*46*). Our study indicates that children who receive an EV-A71 vaccine can still develop HFMD after vaccination, which is a challenge for monovalent EV-A71 vaccines. Even though the EV-A71 vaccine protects against >90% of the EV-A71 infections that occur in children, children still face the risk for infection with other enterovirus serotypes after vaccination. Hence, public health authorities should inform parents and caregivers about the risk for the development of HFMD after EV-A71 vaccination, and multivalent vaccines for HFMD (e.g., EV-A71 combined with other prevalent circulating serotypes CV-A16 and CV-A6) are needed for the HFMD epidemic.

This study has several limitations. First, the burden of HFMD recurrence was underestimated because the disease is substantially underreported in the surveillance system (16%–36% estimated) (*47*) and the observation period for assessing recurrence was insufficient, especially among patients identified in more recent years. Although the recurrence of HFMD is rarely reported in other countries (*48*,*49*), our study suggests that it is not uncommon. Second, because clinical samples were not collected from all patients during each HFMD episode and tested, we could not determine the real number of recurrent HFMD cases; thus, the probabilities of reinfection with an identical enterovirus serotype (i.e., EV-A71 and CV-A16) we calculated might be underestimated. It is not favorable to estimate the number of patients with reinfections of the same serotype because only a small proportion (4.2%) of HFMD episodes have been tested for enterovirus diagnosis, although mathematical modeling methods could be used to solve this problem. This topic requires further exploration. Third, we were unable to describe the features of patients with reinfection of non–EV-A71 and non–CV-A16 serotypes. Fourth, the short interval between consecutive episodes in some patients suggests the potential for co-infections rather than reinfections; thus, co-infections might have occurred and caused a slight overestimation of the recurrence rate for HFMD. However, patients with short intervals between consecutive episodes accounted for a small proportion of the patients with recurrent HFMD, so the effect that co-infections played is relatively limited.

In conclusion, our 8-year surveillance study indicates a high burden of HFMD recurrence among children in China and shows that each episode of recurrent HFMD is more likely caused by a different enterovirus serotype than that of the primary episode (both for patients with EV-A71 and CV-A16 primary infections). Further studies in which virologic diagnosis is performed for all HFMD episodes are needed to better quantify the probability of HFMD recurrence and probability of reinfection by enterovirus serotype, including non–EV-A71 and non–CV-A16 serotypes. Further investigations are also warranted to elucidate the mechanism underlying HFMD recurrences resulting from reinfections with enteroviruses of the same serotype; the protective antibody levels for EV-A71, CV-A16, and other enterovirus serotypes; and the duration of immunity and cross-immunity between serotypes. Finally, more work is needed to study the effect of HFMD recurrence on disease severity, even though no association was observed in this patient cohort.

Technical Appendix Description of identification of patients with hand, foot and mouth disease (HFMD) recurrence, observation periods to find cases of HFMD recurrence, age distribution of patients with recurrent HFMD, and sensitivity analysis of definition for independent episodes.
