# Supplementary material for: Epidemiology of Recurrent Hand, Foot and Mouth Disease, China, 2008–2015
Source: Emerg Infect Dis. 2018 Mar;24(3):432–42. doi: 10.3201/eid2403.171303 (PMC5823341; doi:10.3201/eid2403.171303)
Supplement: Technical Appendix — Description of identification of patients with hand, foot and mouth disease (HFMD) recurrence, observation periods to find cases of HFMD recurrence, age distribution of patients with recurrent HFMD, and sensitivity analysis of definition for independent episodes. [file 17-1303-Techapp-s1.pdf]

Article DOI: https://doi.org/10.3201/eid2403.171303

Epidemiology of Recurrent Hand, Foot and Mouth Disease, China, 2008–2015

Technical Appendix

Identification and Verification of Patients with Hand, Foot and Mouth Disease Recurrence

Two steps were used to screen patients with recurrent hand, foot and mouth disease (HFMD). In step 1, patients with ≥2 records reported in China's national HFMD surveillance system were identified by matching records by any of the following screening criteria: 1) identical identification number and identical or highly similar patient name; 2) identical name of patient's parent, home address, and date of birth and identical or highly similar patient name; and 3) identical contact telephone number, home address, and date of birth and identical or highly similar patient name. The definition for identical or highly similar patient name was as follows: for patient names with 3 Chinese characters, the family name should be identical, and ≥1 character of the given name should be the same; for patient names with 4 Chinese characters, the family name should be identical, and ≥2 characters of the given name should be the same; and for other patient names, all the Chinese characters of the patient names should be identical. Then, we gathered HFMD records matched by any of the previously mentioned 3 screening criteria and gave them a new and identical code to indicate the records belonged to the same patient. In step 2, patients were then checked manually to verify that the reported HFMD episodes had occurred in the same patient. Patients with ≥2 HFMD episodes were verified by meeting 1 of the following criteria: identical Chinese characters or identical pronunciation. Patients were further classified as having recurrence of probable and laboratory-confirmed HFMD.

Technical Appendix Table 1. Distribution of age at first episode of patients with recurrent HFMD, 29 provinces of China, 2008–2015

	Recurrence of laboratory-confirmed HFMD†			Recurrence of probable HFMD		
Age at first episode	No. patients with 2 episodes, N = 1,595, no. (%)	No. patients with 3 episodes, N = 161, no. (%)	No. patients with 4 episodes, N = 11, no. (%)	No. patients with 2 episodes, N = 373,745, no. (%)	No. patients with 3 episodes, N = 21,023, no. (%)	No. patients with ≥4 episodes, N = 1,475, no. (%)
<6 mo	21 (91.3)	2 (8.7)	0 (0)	6,581 (90.4)	644 (8.8)	54 (0.7)
6–11 mo	248 (87.6)	34 (12.0)	1 (0.4)	74,762 (92.3)	5,720 (7.1)	500 (0.6)
1 y	616 (88.5)	74 (10.6)	6 (0.9)	146,242 (93.8)	9,076 (5.8)	655 (0.4)
2 y	379 (90.7)	36 (8.6)	3 (0.7)	79,831 (95.3)	3,748 (4.5)	188 (0.2)
3 y	224 (94.9)	11 (4.7)	1 (0.4)	44,784 (96.7)	1,470 (3.2)	65 (0.1)
4 y	81 (96.4)	3 (3.6)	0 (0)	14,892 (98.0)	305 (2.0)	6 (0.04)
≥5 y	26 (96.3)	1 (3.7)	0 (0)	6,653 (99.0)	60 (0.9)	7 (0.1)

*HFMD, hand, foot and mouth disease.

†Includes recurrent patients who had both laboratory-confirmed and probable HFMD episodes.

Technical Appendix Table 2. Observation period for episodes of recurrence for patients with HFMD, 29 provinces of China, 2008–2015

Observation period after first HFMD diagnosis, mo	No. patients with HFMD recurrence	No. HFMD patients
≥12	383,696	10,253,732
≥24	311,732	7,913,859
≥36	227,160	6,328,740
≥48	149,956	4,509,040
≥60	87,428	3,092,195
≥72	35,750	1,520,812
≥84	9,329	437,827

*HFMD, hand, foot and mouth disease.

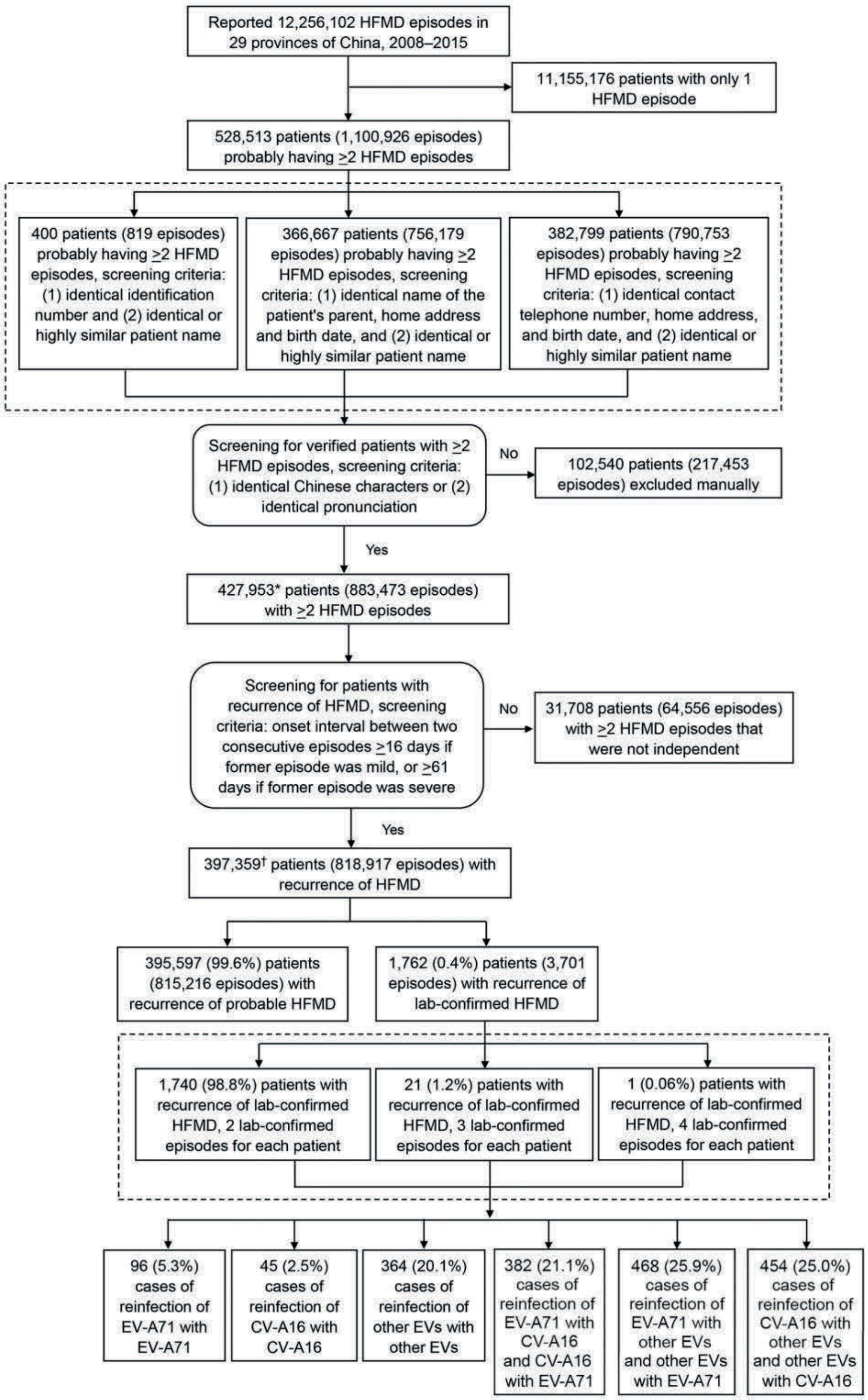

Technical Appendix Figure. Sensitivity analysis of screening for patients with recurrent HFMD from the national HFMD surveillance database, 29 provinces of China, 2008–2015. The interval to define 2 independent episodes was altered to be ≥16 days (for previous mild episodes) and ≥61 days (for previous severe episodes). Percentages do not equal 100% because of rounding. *The number of patients

(427,953) with ≥2 HFMD episodes is higher than expected (528,513 – 102,540 = 425,973) because of improved patient matching. In some situations, the number of patients with ≥2 episodes did not change; for example, a patient initially identified with 3 episodes might have been determined to have only 2 episodes, with the third episode being attributed to a different patient. In other situations, the number of patients with ≥2 episodes decreased; for example, a patient initially identified as having 3 episodes might have been determined to be 3 different patients with 3 different episodes. Therefore, the reduced number of patients (528,513 – 427,953 = 100,560) with ≥2 HFMD episodes is smaller than the number of patients (102,540) excluded manually. †The number of patients (397,359) with recurrence of HFMD is higher than expected (427,953 – 31,708 = 396,245) because some patients needed to be excluded and included. In some situations, patients were completely included or excluded from the recurrent HFMD patient population sample; for example, all 3 episodes of a patient could have been determined to not be independent from each other. In other situations, patients were included and excluded from the recurrent HFMD patient population sample; for example, a patient with 3 episodes might have had 2 episodes that were not independent from each other. In these cases, the patient had 2 episodes included and 1 episode excluded; therefore, the number of included patients plus excluded patients (397,359 + 31,708 = 429,067) exceeded the starting population number (427,953). CV-A16, coxsackievirus A16; EV-A71, enterovirus A71; other EVs, other non–EV-A71 and non–CV-A16 enteroviruses; HFMD, hand, foot and mouth disease.
